# Clinical Aspects and Current Therapeutic Approaches for FOP

**DOI:** 10.3390/biomedicines8090325

**Published:** 2020-09-02

**Authors:** Hiroshi Kitoh

**Affiliations:** Department of Orthopaedic Surgery, Aichi Children’s Health and Medical Center, Obu, Aichi 474-8710, Japan; hkitoh420@gmail.com or hiroshi_kitou@sk00106.achmc.pref.aichi.jp; Tel.: +81-562-43-0500

**Keywords:** fibrodysplasia ossificans progressiva, skeletal malformation, heterotopic ossification, clinical trials

## Abstract

Fibrodysplasia ossificans progressiva (FOP) is an extremely rare heritable disorder of connective tissues characterized by progressive heterotopic ossification in various skeletal sites. It is caused by gain-of-function mutations in the gene encoding activin A receptor type I (*ACVR1*)/activin-like kinase 2 (*ALK2*), a bone morphogenetic protein (BMP) type I receptor. Heterotopic ossification is usually progressive leading to severe deformities in the trunk and extremities. Early clinical diagnosis is important to prevent unnecessary iatrogenic harm or trauma. Clinicians should become aware of early detectable skeletal malformations, including great toe deformities, shortened thumb, neck stiffness associated with hypertrophy of the posterior elements of the cervical spine, multiple ossification centers in the calcaneus, and osteochondroma-like lesions of the long bones. Although there is presently no definitive medical treatment to prevent, stop or reverse heterotopic ossification in FOP, exciting advances of novel pharmacological drugs focusing on target inhibition of the activated *ACVR1* receptor, including palovarotene, REGN 2477, rapamycin, and saracatinib, have developed and are currently in clinical trials.

## 1. Introduction

Fibrodysplasia ossificans progressiva (FOP) is a severely disabling heritable disorder of connective tissues characterized by progressive heterotopic ossification in the skeletal muscles, ligaments, tendons, fascia, and aponeuroses, and malformations of the great toes. Painful recurrent episodes of soft tissue swelling (flare-ups) precede to heterotopic ossification. Flare-ups usually begin in the first decade of life, and several patients with FOP are misdiagnosed as having soft tissue tumors or aggressive fibromatosis before the appearance of heterotopic ossification [1]. They sometimes undergo dangerous and unnecessary diagnostic procedures that provoke heterotopic ossification formation leading to permanent harm and lifelong disability [2]. Early clinical diagnosis and confirmatory genetic testing of FOP are extremely important to prevent additional iatrogenic harm or trauma [3].

Heterotopic ossification throughout the body is progressive, and patient’s disabilities are cumulative [4]. Currently, there are no definitive treatments for FOP; however, there has been substantial recent interest in clinical trials for novel treatments for this specific disease. In this review, we specifically describe various skeletal manifestations suggestive of FOP that can usually be seen before the appearance of heterotopic ossification, to make clinicians aware of these early signs and symptoms of FOP. We also discuss current therapeutic approaches for FOP based on molecular mechanisms of this disease, especially focusing on pharmacological drugs that are currently on-going clinical trials to evaluate their efficacy in FOP patients. Patients’ data presentation including photographs were approved by the ethical committee from the author’s institution. 

## 2. Epidemiology

The worldwide prevalence of FOP is reported to be approximately one in 2 million individuals, with no ethnic, racial, or geographical predisposition [5]. Autosomal dominant transmission with complete penetrance is established, but most cases arise as a result of a spontaneous new mutation [6]. Both genetic and environmental factors affect the phenotype of FOP. A study of monozygotic twins demonstrated that congenital toe malformations were similar within the siblings, but progression of heterotopic ossification varied greatly, suggesting that genetic factors seem to correlate to prenatal development while environmental factors strongly influence postnatal progression of heterotopic ossification [7]. 

## 3. Pathophysiology

FOP is caused by gain-of-function mutations in the gene encoding activin A receptor type I (*ACVR1*)/activin-like kinase 2 (*ALK2*), a bone morphogenetic protein (BMP) type I receptor [8]. Approximately 97% of individuals with FOP carry the recurrent activating mutation (617G>A, R206H) in the *ACVR1*/*ALK2* gene, causing the substitution of a conserved residue in the GS domain of the protein. There are a limited number of patients with other rare mutations in the same gene that may show the unusual clinical features for FOP (FOP variants), most notably greater or lesser severity of the great toe malformations [9,10]. BMPs induce heterotopic bone formation in skeletal muscle in vivo and initiate the differentiation pathway through which myoblasts convert to osteoblastic cells in vitro [11]. BMP receptors (BMPR) belongs to the TGF-β superfamily, and the BMP signaling is initiated with an heteromeric receptor complex consisting of type I (BMPR-I) and type II receptors (BMPR-II). The BMPR-II activates the BMPR-I by transphosphorylating their GS domain, leading to intracellular signaling pathway through phosphorylated SMADs proteins. The mutated *ACVR1* receptor may be constitutive active leading to aberrant signaling through the kinase receptor and overactivation of the downstream SMAD1/5/8 signaling pathway. In addition, mutations appear to change the signaling specificity of the *ACVR1* receptor. The mutated receptor is hyper-responsive to BMP ligands as well as responsive to non-osteogenic ligand, Activin A. Activin A can bind to the mutant *ACVR1* receptor and activate signaling through the SMAD1/5/8 pathway, although it does not activate SMAD signaling when it binds to wild type *ACVR1* receptors [12,13]. Dysregulation of BMP signaling pathway is thought to trigger the formation of the ectopic chondrogenesis, osteogenesis and joint fusion of FOP [14]. To date, all *ACVR1* mutations evaluated for enhanced BMP signaling are gain-of-function mutations [9,15]. 

## 4. Natural Clinical Course

**Figure 1:**
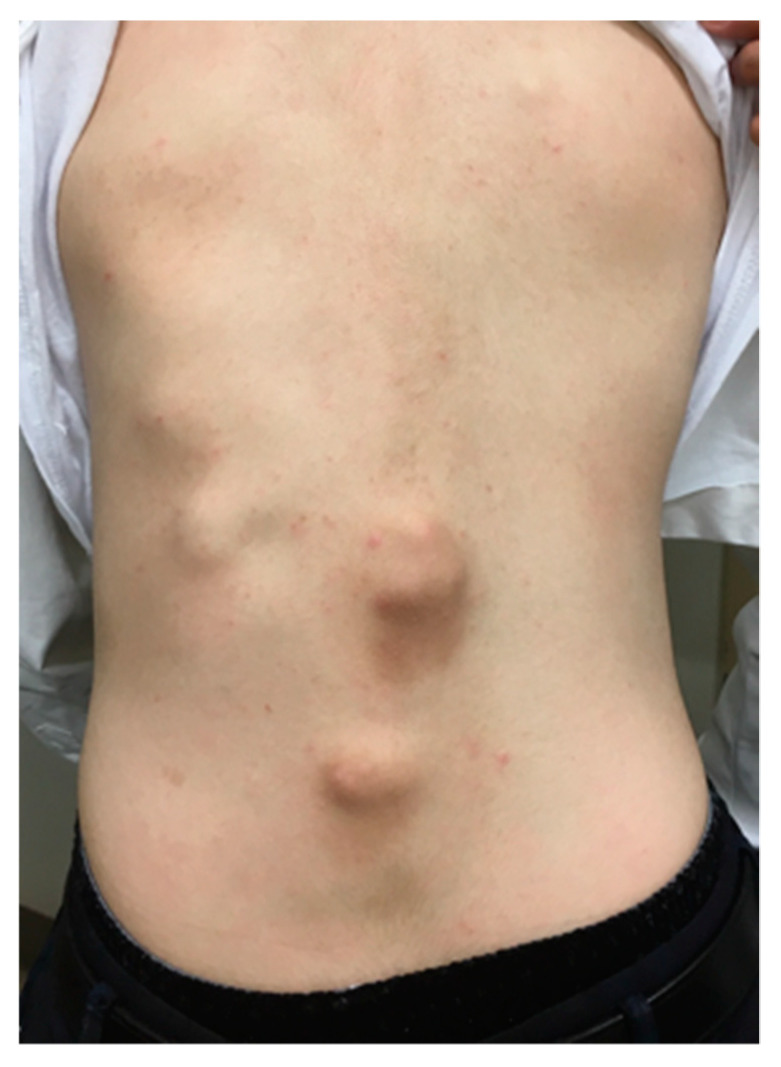
Sixteen-year-old male with fibrodysplasia ossificans progressiva (FOP) demonstrating numerous soft tissue indurations in his back.

**Figure 2:**
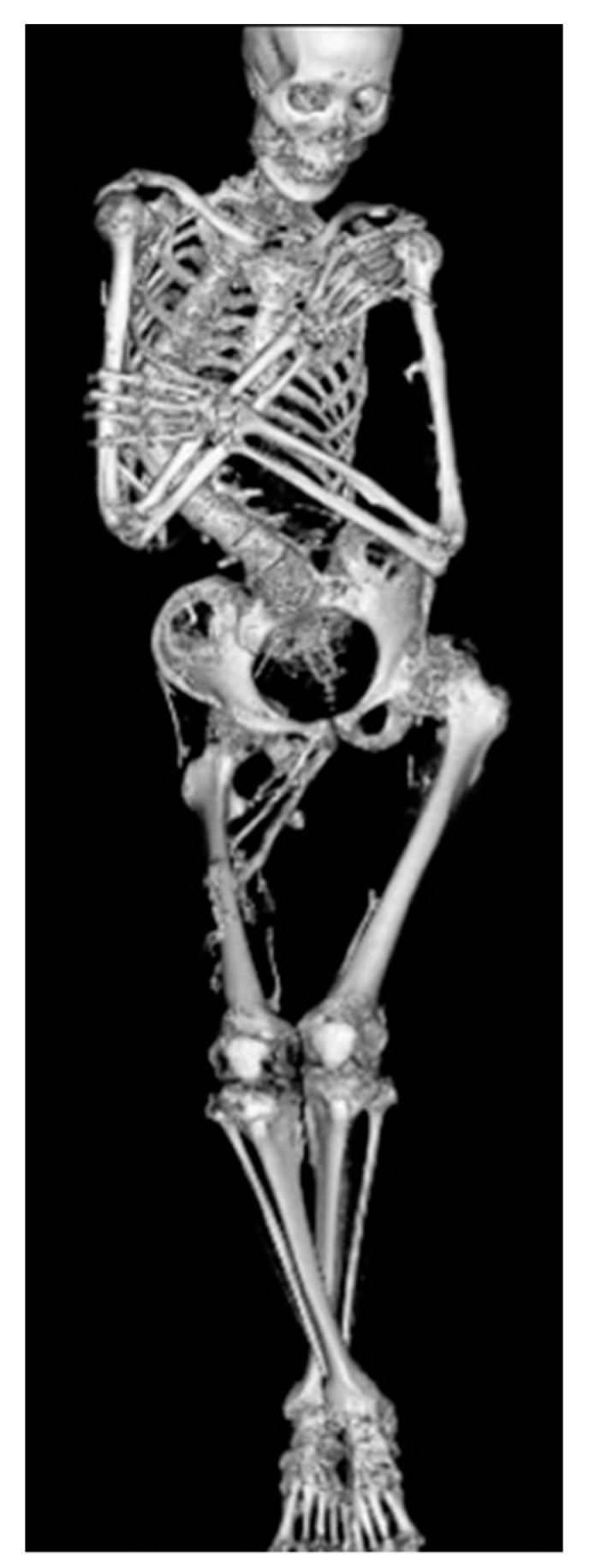
Whole body computed tomography imaging of 26-year-old male with FOP showing severe rigid scoliosis and bilateral hip joint contractures due to heterotopic ossifications across the joints.

Heterotopic ossification in FOP typically begins to form during the first decade of life, with sporadic episodes of flare-ups in the axial skeleton, which are sometimes misdiagnosed as having soft-tissue sarcoma or aggressive juvenile fibromatosis (Figure 1). Flare-ups may occur following a localized invasion mechanism such as trauma, intramuscular injections that lead to bruising, and are occasionally accompanied by sensations of warmth and pain. Traumatic injury and surgical intervention induce explosive new bone formation in FOP. Flare-ups also occur without any causative factor and may even be provoked by systemic inflammation from viral infections such as influenza. Heterotopic ossification progresses in characteristic anatomic and temporal patterns, typically first occurring in the axial, cranial, and proximal regions of the body and later in the appendicular, caudal, and distal regions [16]. Progressive heterotopic ossification throughout the body leads to deformities in the trunk and joint contractures in the extremities (Figure 2). Oddly, the disease seems to spare some anatomical locations, including the ocular muscle, tongue, cardiac muscle, and diaphragm. Arm function reflects early decreases in the activity of daily living [17]. The process of heterotopic ossification is highly individualized. Systemic ankyloses result in difficulty in walking and respiratory dysfunction as the disease progresses in some patients. The patient’s age is correlated with functional disability evaluated by patient reports, as well as the volume of heterotopic ossifications [18]. Most patients are confined to a wheelchair by the third decade of life, and require lifelong assistance in performing activities of daily living [19]. Heterotopic ossification in the temporomandibular joint and surrounding areas often results in trismus which interferes with eating and leads to severe weight loss. Heterotopic ossification of the spine and thoracic cage may cause rigid fixation of the chest wall and respiratory dysfunction. The median age at death is approximately 40 years, but the median estimated life expectancy is 56 years [20]. Death often results from complications of thoracic insufficiency syndrome or pneumonia [21]. The overall prognosis for this disease is considered poor. 

## 5. Skeletal Malformations 

**Figure 3:**
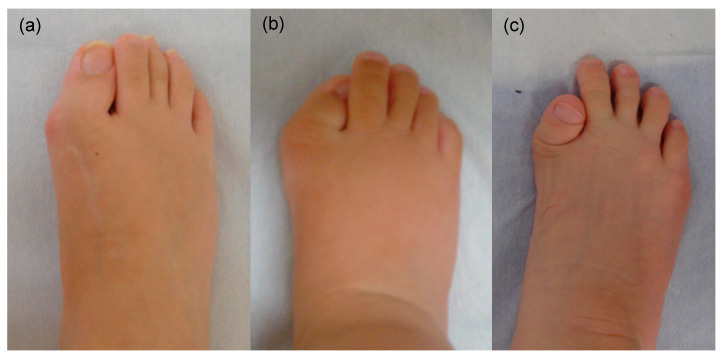
Gross appearance of the right foot in FOP patients from 18-year-old female (**a**), 10-month-old male (**b**), and 7-year-old male (**c**). The degree of hallux valgus and shortening of the great toes are variable.

**Figure 4:**
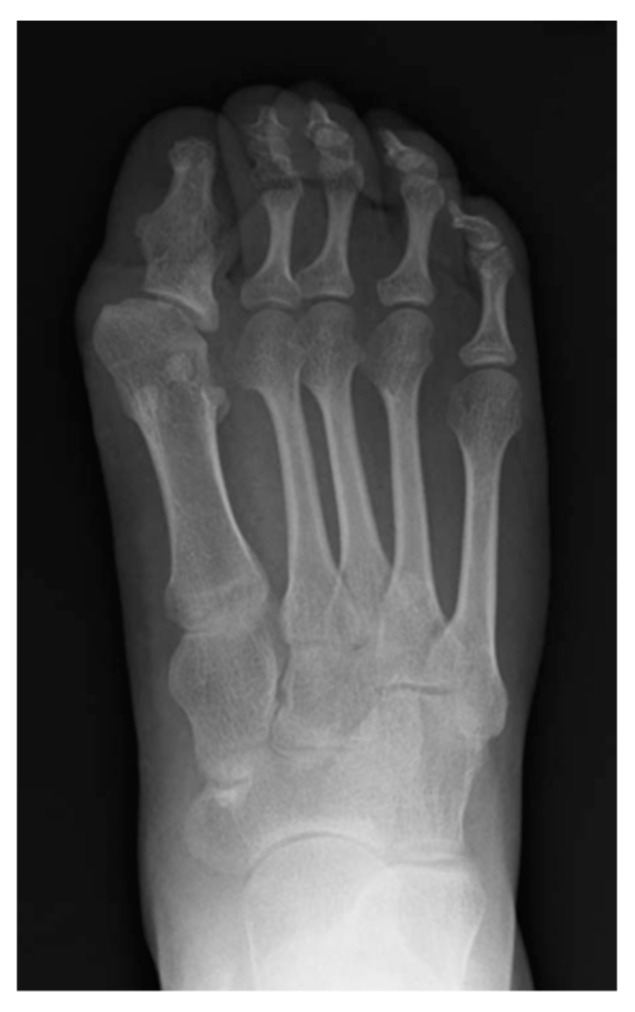
Anteroposterior radiograph of the right foot of 20-year-old female with FOP demonstrating medially deviated metatarsal bone and fused proximal and distal phalanges.

Individuals with FOP appear normal at birth, but there are a variety of congenital skeletal malformations. Deformities of the great toes are well-known and are the most prevalent indicators of this disorder. A shortened great toe and hallux valgus are characteristically found before the appearance of heterotopic ossification. The tip of the great toe usually locates proximal to the distal interphalangeal joint of the second toe. The degree of hallux valgus and shortening of the great toe varies among the feet in gross findings (Figure 3). Radiologically, the proximal phalanx is consistently shortened and sometimes shows triangular shape. The metatarsal bone is also shortened and sharpened to the medial side, deviating the proximal phalanx laterally from the metatarsal axis [22]. Fusion between the proximal and distal phalanx is observed with advancing age (Figure 4). Although the common *ACVR1* mutation (R206H) shows a homogeneous phenotype of the great toes, several atypical mutations have been identified in patients who showed normal-appearing great toes or severe truncation deformities of digits [9,23,24]. BMPs exert an anti-chondrogenic effect on early limb bud mesenchymal cells [25]. The differences in genotype of the *ACVR1* may be related to the strength of the anti-chondrogenic effect on condensing mesenchymal cells via BMP signaling, leading to variety of phenotype in great toes. 

**Figure 5:**
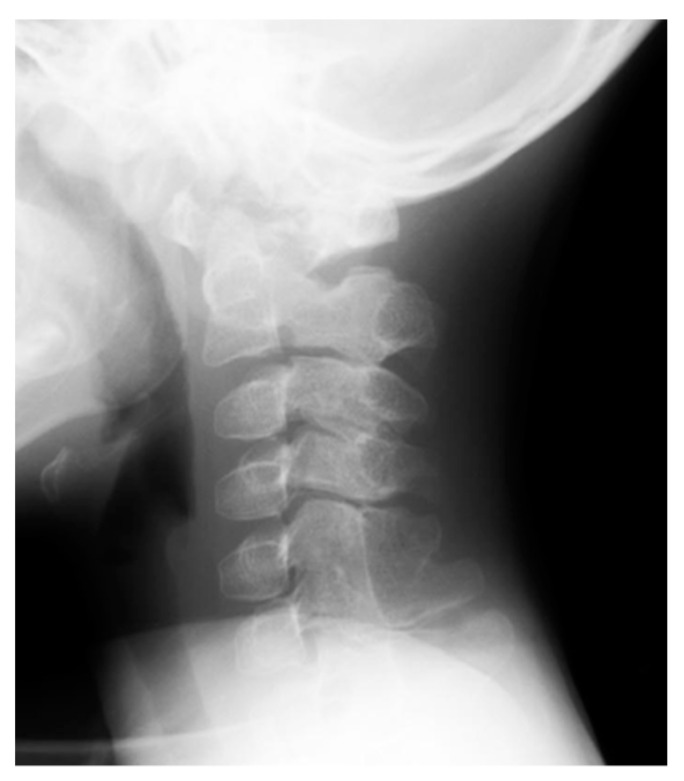
Lateral radiograph of the cervical spine of a FOP boy at the age of 7 years showing hypertrophy of the laminae and spinous processes and complete osseous fusions in facet joints and spinous processes between C5 and C6.

Stiffness of the neck is an important early clinical sign of FOP in infants and it can precede the appearance of heterotopic ossification at that site. Crawling is often disturbed due to limited neck extension. Radiologically, enlarged posterior elements of the cervical spine, including pedicles, laminae, and spinous processes, are characteristic. Vertebral bodies are tall and narrow [26,27]. The cervical spine often becomes ankylosed resulting from fusion of the facet joints early in life (Figure 5). Using the chick embryos, genetically-engineered overexpression of BMP2/4 both dorsally and laterally to the neural tube manifested combined phenotypes of hypertrophic spinous processes and large deletion of the lateral and ventral parts of vertebral bodies [28]. Mesenchymal condensations at the paraxial mesoderm in FOP, where BMP signaling is aberrantly activating, could be responsible for both enlarged spinous processes and relatively tall vertebral bodies. 

**Figure 6:**
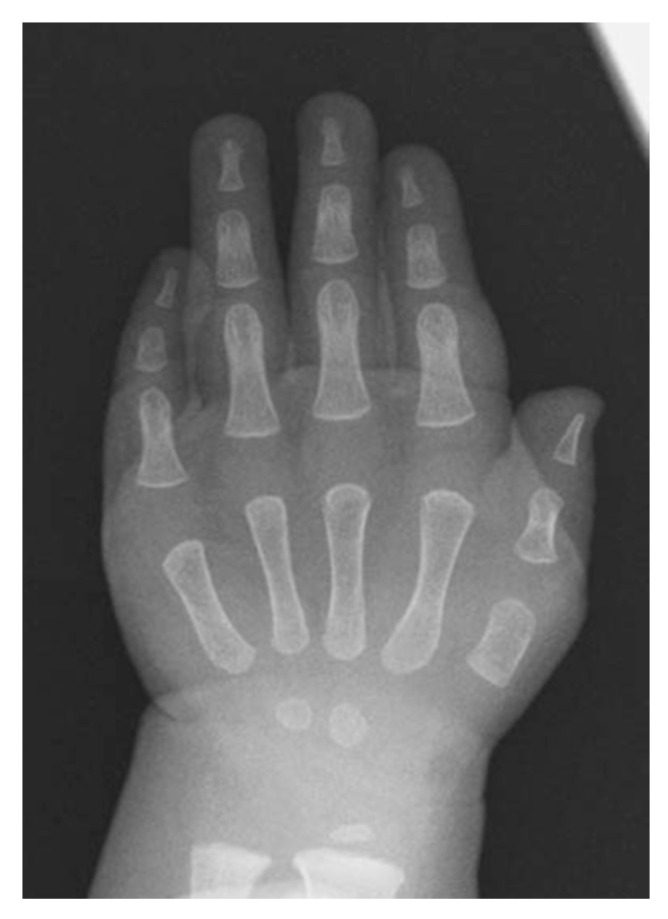
Anteroposterior radiograph of the left hand of a FOP boy at the age of 11 months demonstrating marked shortening of the first metacarpal bone.

Short thumb is another clinical feature of FOP. It is mainly due to shortening of the first metacarpal bones. Quantitative radiological examinations demonstrated shortened distal phalanx relative to the second metacarpal bone and disproportionate shortening of the first metacarpal bone [29] (Figure 6). The thumb is the last digit in the autopod to form, and it is different from other digits in terms of its relative position, shape, size, and number of phalanges. These unique thumb identities may be attributed to the expression profile of *HoxD* genes, which are pivotal transcriptional factors regulating limb patterning and growth [30]. *HOXD10* to *D13* genes are expressed in the future digit II-V area in the autopod during the hand plate formation, whereas the sole expression of the *HOXD13* gene in the presumptive digit I area is of great significance [31]. Interestingly, BMP signaling-dependent SMAD1/4 proteins prevented *HoxD10* and *HoxD13* from binding to DNA targets [32]. Mesenchymal condensation and chondrocyte proliferation of the presumptive thumb area could be regulated by direct interactions between BMP-induced *SMADs* and *HoxD13*. Dysregulated BMP signal transduction during embryogenesis may cause the relative shortening of the first metacarpals and distal phalanges of the thumb in FOP. 

**Figure 7:**
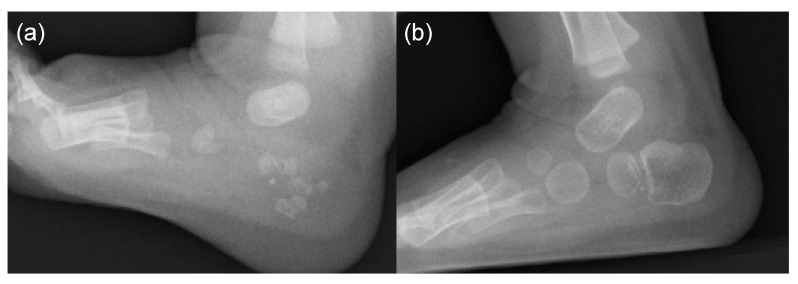
Lateral foot radiographs of a FOP boy at 3 months of age (**a**) and 11 months of age (**b**). Punctate multiple ossifications in early infancy gradually changed to double ossification centers.

We have demonstrated distinctive multiple ossification centers and plantar spurs in the calcaneus in some FOP infants [33]. These findings were bilateral and symmetrical. Multiple (or punctate) calcaneal ossifications are seen in early infancy, which evolved into double ossifications and finally completely coalesced with age (Figure 7). Similar duplicate calcaneus is observed in an infant with Larsen syndrome, which is caused by heterozygous mutations in the filamin B gene (*FLNB*). *FLNB* mutant mice display ectopic mineralization in various cartilaginous elements, but those on a *Runx2* haploinsufficiency background show a completely or partially rescued phenotype, indicating mutated *FLNB* interacts with Runx2-TGFβ-SMADs pathway [34]. Molecular interactions between *FLNB* and *SMADs* signaling in skeletal morphogenesis may lead to similar phenotypes of ossifications in the calcaneal region in Larsen syndrome and FOP. Calcaneal spurs are pedunculated and projected posteriorly in early infancy, and they become sessile and finally smaller in size with age. The normal calcaneal spur is morphologically indistinguishable from the late manifestation of the calcaneal spur in FOP, but the early pedunculated appearance in FOP is not seen in the normal spur. 

**Figure 8:**
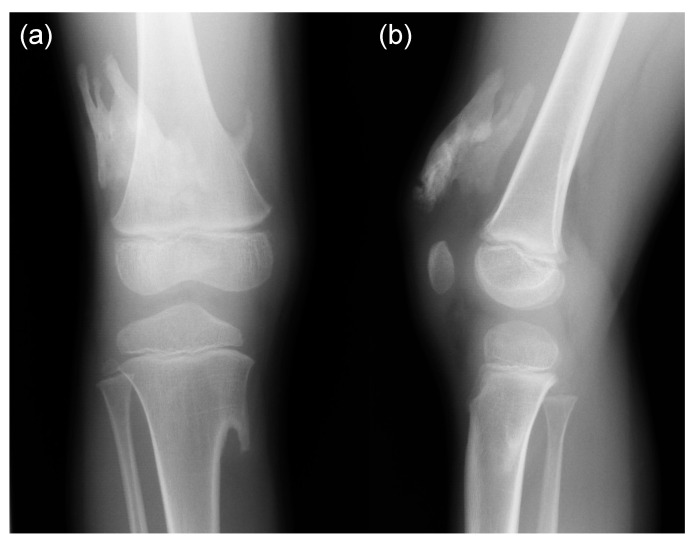
Anteroposterior (**a**) and lateral (**b**) knee radiographs of the FOP boy at the age of 5 years showing heterotopic ossification at the distal thigh and osteochondroma-like lesions of the distal femur and proximal tibia.

Broad femoral necks with metaphyseal widening and osteochondroma-like lesions in the metaphysis of the of the long bones are also early findings in FOP infants. In addition to osteochondroma-like lesions, heterotopic ossification around the knee should not be misdiagnosed as soft tissue tumor (Figure 8). Osteochondroma-like lesions are commonly observed in multiple hereditary exostoses, which is caused by mutations in *EXT1*, *EXT2*, or *EXT3* genes, encoding tumor suppressors and glycosyltransferases involved in the biosynthesis of heparan sulfate proteoglycans (HSPGs) [35]. HSPGs bind to and modulate the activity of Indian hedgehog (Ihh), which is expressed in prehypertrophic chondrocytes and regulates chondrocyte maturation, and the abnormal modulation of the tightly regulated Ihh/parathyroid hormone related peptide (PTHrP)-negative feedback loop has been proposed as a molecular model of osteochondroma formation in multiple hereditary exostoses [36]. Constitutive active *ACVR1* R206H mutation resulted in dramatic upregulation of Ihh at the perichondrium and a delay in chondrocyte differentiation in a chicken limb bud model [37]. Thus, osteochondroma formation in FOP could be mediated by disruption of the BMP/Ihh/PTHrP-negative feedback loop at the perichondrium. These skeletal abnormalities suspicious of FOP in infants can lead to early clinical diagnosis, confirmatory diagnostic genetic testing, and the avoidance of iatrogenic harm or trauma. 

## 6. Managements and Treatments

There is presently no definitive medical treatment to prevent, stop or reverse heterotopic ossification in FOP. Avoidance of trauma and prevention of injury remain the mainstays of therapy. Surgical removal of heterotopic ossification often leads to significant recurrence and expansion of ossification. Bracing for spinal deformity is ineffective [38]. Restriction of activity may be helpful to reduce trauma, but compromise of independence may be unacceptable to patients as well as their parents. Physical rehabilitation to maintain joint mobility may be harmful by provoking or exacerbating lesions and it should be focused on enhancing activity of daily living through approaches that avoid a passive range of motion exercises. Occupational therapy and vocational education consultations may be useful. Overstretching of the jaw and intramuscular injections of local anesthesia should not be attempted in dental care. A locked jaw sometimes necessitates surgery to avoid life threatening complications. Since conductive hearing loss is common, children should have audiology evaluations regularly. The management of FOP requires education of patients and caregivers, the use of medications to settle inflammation and flare-ups, instructions to ensure proper oral care, and other compensatory approaches that aid in rehabilitation [39]. 

The use of short-term high-dose corticosteroids is based on its potent anti-inflammatory effects [40]. It may help reduce the intense inflammation and tissue edema when they are used in an early stage of flare-ups. They can relieve but not completely resolve symptoms of flare-ups [41]. Corticosteroids are most effective if used within the first 24 h of a new flare-up. The dose of corticosteroids is dependent on body weight, and a recommended dose of prednisone for acute flare-ups is 2 mg/kg/day, administered as a single daily dose for no more than 4 days. Corticosteroids should be used for treatment of flare-ups that affect major joints, the jaw, or the submandibular area, and should not be used for flare-ups that involve the back, neck, or trunk due to the long duration and recurring nature of these flare-ups. Corticosteroids should not be used for long-term, and when prednisone is discontinued, non-steroidal anti-inflammatory drugs (NSAIDs) or selective cyclooxygenase-2 (COX2) inhibitors may be used for the duration of flare-ups, although there is no evidence that chronic treatment with these drugs prevent flare-ups in FOP. Bisphosphonates have been used for the symptomatic management of flare-ups in FOP, although concrete clinical data for these treatments are sparse [42]. Mast cells could provide an important role for the pathology of heterotopic ossification in FOP [43]. Imatinib, a tyrosine kinase inhibitor initially developed for chronic myeloid leukemia, has anti-proliferative and immunomodulatory effects in mast cells. The administration of imatinib demonstrated positive effects on decrease in the intensity of flare-ups in seven FOP patients who did not respond the standard medications such as corticosteroids, NSAIDs, or intravenous bisphosphonates [44]. 

## 7. On-Going Clinical Trials for FOP (Phase 2 or Phase 3)

**Figure 9:**
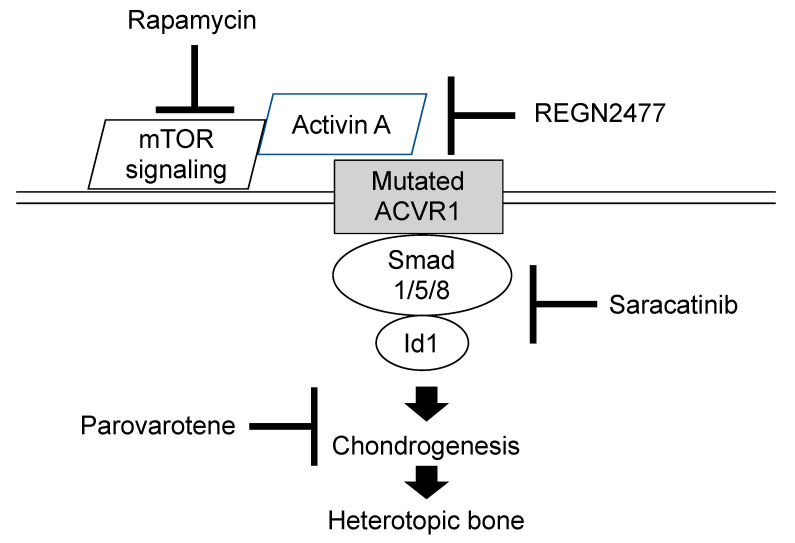
Molecular targeting of therapeutic drugs on on-going phase 2 or 3 clinical trials.

Several researches to develop therapeutic drugs have focused on target inhibition of the *ACVR1* receptor, *ACVR1* ligand, BMP intracellular signaling, and inflammatory triggers of disease activity. Exciting advances in new therapeutic approaches for FOP have developed recently [45,46,47]. We highlight novel treatment drugs that are currently on-going phase 2 or phase 3 clinical trials (Figure 9). 

Retinoid signaling is normally attenuated during chondrogenesis and exogenous retinoid agonists can block chondrogenesis effectively and rapidly [48]. Agonists for retinoic acid receptors (PARα or RARγ) experimentally inhibited chondrogenesis of heterotopic ossification in transgenic mice model of FOP, and the RARγ agonists were far more effective [49]. One of the RARγ class drugs is palovarotene, a highly specific RARγ agonist that has already been evaluated in another clinical trial for α-1-antitrypsin-induced emphysema, and its safety profile has been well-characterized. Palovarotene inhibits heterotopic ossification and maintains limb mobility and growth in mice model of FOP [50]. Palovarotene is also evaluated in another phase 2 trials for treatment of hereditary multiple exostoses to suppress the formation of osteochondromas. Phase 2 clinical trials were initiated in 2014 by Clementia Pharmaceuticals to evaluate the safety and efficacy of palovarotene for treatment of FOP (Clinicaltrials.gov registration NCT02190747). The primary outcome was to compare the volume of heterotopic ossification formation between treated patients and untreated patients. Palovarotene decreased the percentage of FOP patients who develop heterotopic ossification, the time to flare-ups resolution, and patient-reported pain. Phase 3 trial is currently in progress (Clinicaltrials.gov registration NCT03312634). Palovarotene is a known teratogen that causes limb malformations in the developing fetus and may decline growth in children [51]. Other potential risks of palovarotene include pancreatitis, hearing and vision impairment, mouth ulcer, sensitivity to sunlight, and dry skin. These adverse events are being monitored closely during the trials. 

The R206H mutation causes the *ACVR1* receptor to misinterpret activin A and generate a signal as if BMP ligands are present [12]. The *ACVR1* mutant mice developed more heterotopic ossification throughout the skeleton when activin A was injected, and those treated with a blocking antibody of activin A did not develop heterotopic ossification [48]. Activin A is, thus, an obligatory secreted factor that is required for the initiation of heterotopic ossification in FOP, and the blocking of activin A could prevent the formation of heterotopic bone. As a result of preclinical studies, REGN 2477 (garetosmab)—an antibody that binds to activin A and blocks its activity—is now in a clinical trial to examine safety, tolerability, and efficacy on abnormal bone formation in adult patients with FOP (Clinicaltrials.gov registration NCT03188666).

Activin A enhances the chondrogenesis of induced mesenchymal stromal cells derived from FOP patients-derived induced pluripotent stem cells (FOP-iPSCs) via the aberrant activation of BMP signaling in vitro, and induced endochondral ossification of FOP-iPSCs in vivo [52]. By using a high-throughput screening system of small molecules to suppress activin A induced chondrogenesis, Hino et al. demonstrated that mTOR signaling is a critical pathway for the aberrant chondrogenesis of mesenchymal stromal cells derived from FOP-iPSCs and inhibited the heterotopic ossification of multiple model mice, including FOP-ACVR1 transgenic mice and a heterotopic ossification model utilizing FOP-iPSCs [53]. Rapamycin is a commonly-used immunosuppressant that exerts its biological effect by inhibiting mTOR1 kinase activity. Heterotopic ossification was decreased after treatment with rapamycin in mice model of FOP as well as FOP-iPSC-based heterotopic ossification model mice [54]. A phase 2 clinical trial for a 6-month randomized placebo-controlled study and subsequent open label extension study is now opening in Japan (UMIN000028429). Primary endpoint for evaluating the efficacy of rapamycin is based on objective physical function assessment using the Japanese version of Health Assessment Questionnaire or Childhood Health Assessment Questionnaire. 

Saracatinib, also known as AZD0530, is an investigational drug that was initially developed as a potential treatment for patients with cancer. Saracatinib inhibits the serum activation of Id1, which is a transcriptional factor mediated by Smad 1/5/8 phosphorylation, by direct inhibition of BMPR-I kinase activity [55]. Other research also demonstrated that Saracatinib was effective at suppressing the enhanced chondrogenesis of FOP-iPSCs and suppressed the heterotopic ossification or bone formation in multiple FOP animal models [49]. A phase 2A proof of concept study including a 6-month randomized placebo-controlled study and 12-month open label extension study using historical data is proposed in the Netherlands, the United Kingdom, and Germany (Clinicaltrials.gov registration NCT04307953). 

## 8. Conclusions

Clinicians should become aware of early detectable skeletal malformations, including great toe deformities, shortened thumb, neck stiffness associated with hypertrophy of the posterior elements of the cervical spine, multiple ossification centers in the calcaneus, and osteochondroma-like lesions of the long bones, to make an early diagnosis and prevent iatrogenic harm or trauma. Although there is presently no definitive medical treatment to prevent, stop or reverse heterotopic ossification in FOP, exciting advances in novel therapeutic approaches using pharmacological drugs, including palovarotene, REGN 2477, rapamycin, and saracatinib, have been developed and are currently in clinical trials.
